# Malaria Diagnosis in Non-Endemic Settings: The European Experience in the Last 22 Years

**DOI:** 10.3390/microorganisms9112265

**Published:** 2021-10-31

**Authors:** Adriana Calderaro, Sara Montecchini, Mirko Buttrini, Giovanna Piccolo, Sabina Rossi, Maria Cristina Arcangeletti, Benedetta Farina, Flora De Conto, Carlo Chezzi

**Affiliations:** 1 Department of Medicine and Surgery, University of Parma, Viale A. Gramsci 14, 43126 Parma, Italy; sara.montecchini@unipr.it (S.M.); mirko.buttrini@unipr.it (M.B.); giovanna.piccolo@unipr.it (G.P.); mariacristina.arcangeletti@unipr.it (M.C.A.); benedetta.farina@studenti.unipr.it (B.F.); flora.deconto@unipr.it (F.D.C.); carlo.chezzi@unipr.it (C.C.); 2 Unit of Clinical Microbiology, University Hospital of Parma, Viale A. Gramsci 14, 43126 Parma, Italy; srossi@ao.pr.it

**Keywords:** imported malaria, diagnosis, molecular methods, Europe, polymerase chain reaction

## Abstract

Accurate, prompt, and reliable tools for the diagnosis of malaria are crucial for tracking the successes or drawbacks of control and elimination efforts, and for future programs aimed at global malaria eradication. Although microscopy remains the gold standard method, the number of imported malaria cases and the risk of reappearance of autochthonous cases stimulated several laboratories located in European countries to evaluate methods and algorithms suited to non-endemic settings, where skilled microscopists are not always available. In this review, an overview of the field evaluation and a comparison of the methods used for the diagnosis of malaria by European laboratories is reported, showing that the development of numerous innovations is continuous. In particular, the combination of rapid diagnostic tests and molecular assays with microscopy represents a reliable system for the early diagnosis of malaria in non-endemic settings.

## 1. Introduction

The genus *Plasmodium* consists of over 200 widely distributed species, of which at least six regularly infect humans: *Plasmodium falciparum* (*Pf*), *P. vivax* (*Pv*), *P. malariae* (*Pm*), *P. ovale wallikeri* (*Pow*), *P. ovale curtisi* (*Poc*), and *P. knowlesi* (*Pk*) [1]. However, recently, cases of susceptibility to the non-human primate *Plasmodia,* such as *P. cynomolgi* in Southeast Asia and *P. brasilianum* and *P. simium* in South America, have been described [2,3,4].

Among species causing malaria in humans, *P. falciparum* and *P. vivax* pose the greatest threat: in 2018 *P. falciparum* accounted for 99.7% of estimated cases in the World Health Organization (WHO) African regions; and *P. vivax* is the most common species in the WHO regions of Americas, accounting for 75% of infections [5].

Malaria is a febrile illness and clinical symptoms of uncomplicated malaria include fatigue, headaches, muscle aches, malaise, abdominal discomfort, fever, nausea, and vomiting [6]. Specific diagnostic methods are needed to differentiate between malaria and other febrile illnesses. An early diagnosis can prevent further progression and lower the severity of the disease, especially for children under 5 years of age who accounted for about 67% of deaths in 2018 due to severe malaria worldwide [5]. For the most effective treatment of malaria, it is important to know the species of *Plasmodium* interested and the parasitic burden in the blood. Parasite count is mandatory in cases of infection with *P. falciparum*, because it is one of the criteria used to define severe malaria (parasitemia >4% in adults and >10% in children). Different patient management modalities are applied if the parasitemia is >2% [7]. The presence of mature asexual forms (>20% of parasites) is another criterion for the definition of severe *P. falciparum* malaria [6,7]. 

Accurate, prompt, and affordable diagnostic tools are also pivotal for tracking the successes or drawbacks of control and elimination efforts, and for future programs aimed at global malaria eradication. Active surveillance of the disease in each geographical area is essential for a program to succeed. The WHO Global Technical Strategy for Malaria aims, by 2030, to reduce malaria case incidence and mortality rates by 90%, compared to the 2015 baseline, to interrupt malaria transmission in at least 35 countries and to prevent its re-establishment in all malaria-free countries. The aim of surveillance is to detect all malaria infections and to investigate each individual case of infection, to differentiate imported cases, namely, infections acquired outside the areas in which they are diagnosed, from those acquired locally [8,9]. 

In fact, with 229 million cases and 409,000 deaths, especially among children (estimated in 2019), malaria is one of the most severe public health problems worldwide [5,10]. Although it occurs mostly in poor tropical and subtropical areas of the world [5,10], a high number of cases are also reported in non-endemic settings, such as Europe, where it is a medical emergency. 

Malaria is thought to have arrived in South Europe via the Nile Valley during the Neolithic period, from whence it has been spread to the entire continent, where it remained endemic for more than 2000 years until its elimination by 1978 [11]. During 2011–2012, outbreaks were reported in an agricultural area of South Greece, and sporadic locally acquired cases were recorded throughout the country [12]. During 2019, the European Centre for Disease Prevention and Control (ECDC) reported 8641 malaria cases in the EU/EAA (99% confirmed). Among the episodes with known importation status, 99.8% were travel-related. Nine confirmed cases were reported as acquired in the EU (2 in Germany, 2 in Greece, 2 in Spain, 2 in France, and 1 in the Netherlands) [13]. 

The consistent number of imported malaria cases and the risk of reappearance of autochthonous cases stimulated several laboratories located in European countries to evaluate methods and algorithms for the diagnosis that are best suited to non-endemic settings, where skilled microscopists are not always available, especially when the diagnosis is required in emergencies outside laboratory opening hours [10,14,15].

In this review, an overview of the studies performed in the period 1999–2021 by European laboratories, concerning the evaluation and the comparison of methods for the diagnosis of malaria, was reported.

## 2. Gold-Standard Method

**Figure 1:**
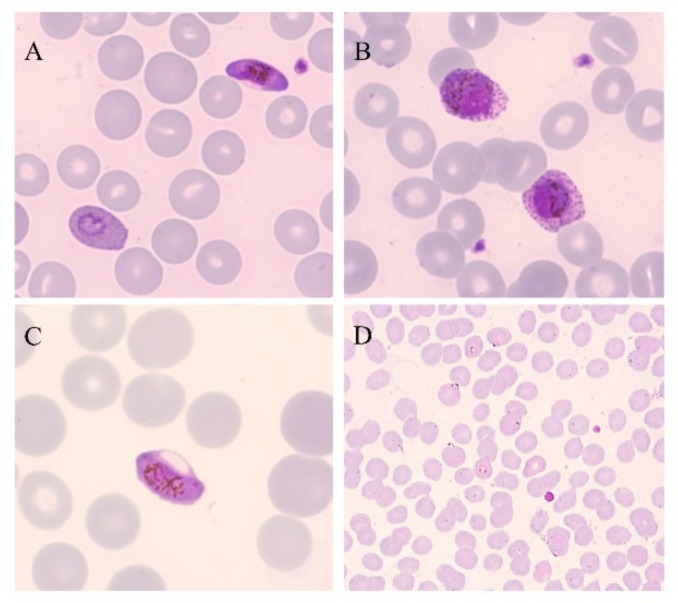
Thin blood smears of blood samples from malaria cases prepared and stained with Giemsa. (**A**) *P. falciparum* gametocyte and *P. ovale* trophozoite (100×). (**B**) *P. ovale* gametocytes (100×). (**C**) *P. falciparum* gametocyte (100×). (**D**) *P. falciparum* trophozoites (40×). (Picture by A. Calderaro, Department of Medicine and Surgery, University of Parma, Parma, Italy).

**Figure 2:**
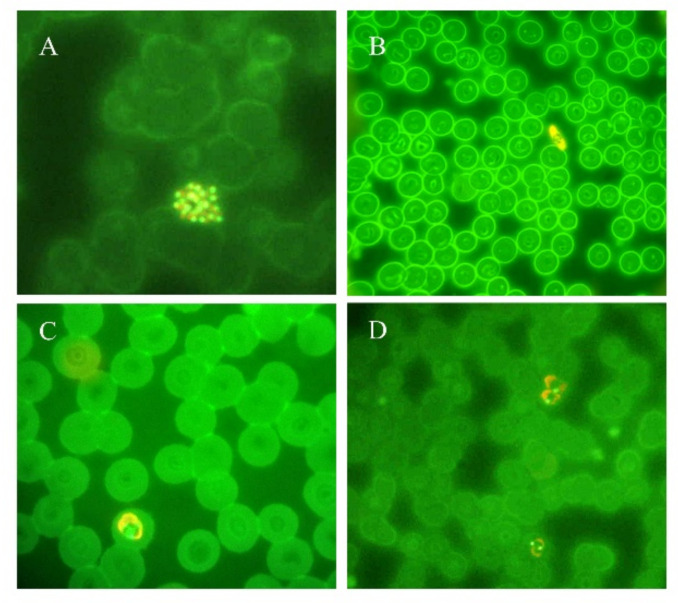
Thin blood smears of blood samples from malaria cases prepared and stained with acridine orange. (**A**) *P. vivax* schizont (100×). (**B**) *P. falciparum* gametocyte (40×). (**C**) *P. ovale* trophozoite (100×). (**D**) *P. vivax* trophozoites (100×). (Picture by A. Calderaro, Department of Medicine and Surgery, University of Parma, Parma, Italy).

Microscopic examination of blood films was the first technique used, and remains the “gold standard” and the most widely used method for the diagnosis of malaria [16,17]. Thick and thin blood smears stained with Giemsa, Wright’s, or Field’s allows to rapidly detect and differentiate, when possible, the various species and the parasite stages, and quantify the parasite density, known as parasitemia (Figure 1) [16,17,18]. Thick blood film is a concentration technique that provides enhanced sensitivity in case of low level parasitemia [18]. Stained thin blood film is less sensitive; however, it is the most used technique for the diagnosis of malaria and for the parasitemia determination because the organisms are easier to see and count [16,18]. The sensitivity and specificity for this method are 95% and 98%, respectively, when the polymerase chain reaction (PCR) is used for comparison; the limit of detection for this method is approximately 50–200 parasites per μL of blood [19]. To enhance the detection of *Plasmodia* in blood film, alternative methods can be used in areas where training and expensive equipment can be introduced, such as staining with fluorescent dyes having affinity for the nucleic acid (especially acridine orange and benzothiocarboxypurine) (Figure 2) directly on blood smears or using quantitative buffy coat (QBC), a concentration method associated with fluorescent staining [16,18,20]. 

Overall, microscopic examination provides rapid and inexpensive detection and identification of *Plasmodia* at the species and stage levels, and allows their quantification in peripheral blood in order to monitor patients with malaria, including follow-up during specific therapy. It is noteworthy that microscopy requires specific skills rarely available in non-endemic settings, especially when cases of mixed or sub-microscopic infection occur [16]. Although microscopy remains the gold standard method, most of the laboratories located in non-endemic countries evaluated further techniques that can be used for malaria diagnosis.

## 3. Rapid Diagnostic Tests

Rapid diagnostic tests (RDT) are immunochromatographic assays for quickly (15–20 min) establishing the diagnosis of malaria infection by detecting specific malaria antigens in blood [17,18]. The first commercial RDT was distributed in 1994 to improve the diagnosis of malaria, particularly in endemic remote areas, and since then more than 200 devices have been marketed [21,22].

**Figure 3:**
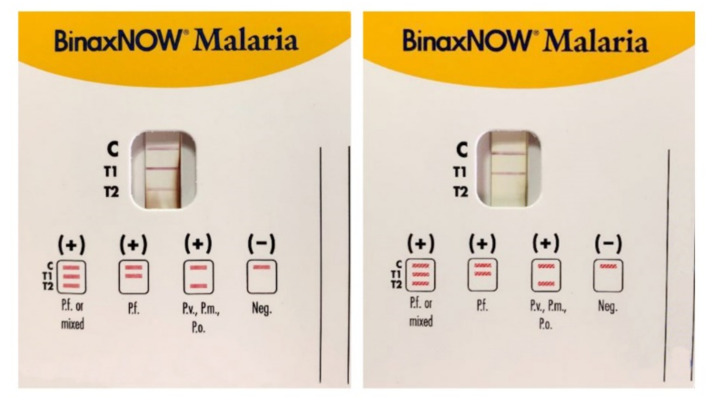
Immunocromatographic assay for the search of *Plasmodia* antigens in blood samples: *P. falciparum* (*Pf*), *P. malariae* (*Pm*), *P. vivax* (*Pv*), and *P. ovale* (*Po*). C is the control band, T1 band corresponds to *P.falciparum* histidine-rich protein 2 (HRP2), and T2 band corresponds to parasite lactate aldolase. A *Pf* or mixed infection on the left and a *Pf* infection on the right. (Picture by A. Calderaro, Department of Medicine and Surgery, University of Parma, Parma, Italy).

The availability of commercial kits (Figure 3) providing all the necessary reagents and their ease of performance and interpretation have made them an increasingly common tool to support microscopy in non-endemic areas where the low prevalence of malaria does not give the microscopists the chance to maintain their interpretation skills [16,18].

The antigens currently used in RDTs available are *Plasmodium falciparum*-specific histidine-rich protein 2 (HRP2), *Plasmodium* pan-specific lactate dehydrogenase (pLDH), and pan-malarial aldolase for *Plasmodia* infecting humans [23]. HRP2 was the first antigen selected to develop an RDT because of its abundance in *P. falciparum*: it is produced by asexual stages and gametocytes of such *Plasmodium*, and it is expressed on red blood cells’ (RBCs) surface. pLDH is expressed at high level in asexual stages of *P. falciparum*, *P. ovale*, *P. vivax*, and *P. malariae* human malaria parasites. Aldolase is a pan-specific enzyme involved in the glycolytic pathway of the malaria parasites [18]. The limit of detection of RDTs is approximately 200–2000 parasites per μL of blood [24].

Several European laboratories evaluated the performance and/or the usefulness of commercial RDTs for their inclusion in the malaria diagnosis workflow, as reported in Table 1 [15,21,25,26,27,28,29,30,31,32,33].

Different performances among the commercial assays were observed; however, overall, the authors concluded that RDTs are useful supporting tools for the diagnosis of malaria in non-endemic settings. Though they cannot be considered as unique diagnostic methods, these tests help the operator to achieve a rapid and easy to perform interpretation, especially if a trained microscopist is not always available; this can avoid delay in the management of life-threatening malaria cases [12,15,25,26,27,28,31,32]. However, if a negative result is obtained, the disease cannot be ruled out [12,25]. As expected, false-positive and false-negative results were observed. Concerning *P. falciparum* malaria, the false-negative results observed can be attributed to a low level of parasitemia that appears to be critical for this assay [26]. Furthermore, mutations/deletions in HRP2 gene have been reported to affect the results of RDTs based on the detection of this antigen [25,26,31]. In some cases, a prozone effect could be the explanation of false-negative results, although not observed in the studies reported above [15,31]. False-positive results could have various explanations. Although rarely, a cross-reaction with rheumatoid factor can occur. More frequently, in the case of HRP2 based assays, the antigen can persist for weeks following the eradication of the asexual-stage parasitemia [31,32] because of the delayed clearance of circulating antigen and because of the persistence of sexual-stage forms producing antigen [31]. On the contrary, pLDH is produced only by viable parasites; it is detected earlier than HRP2 and it appears to be cleared from the bloodstream within 24 h of a treatment [31,32]. However, it cannot be ignored that microscopy, despite being the reference test, could result negative when asexual-stage parasitemia runs at a level below its detection limit, and a related result by RDT could be misinterpreted as a false positive [31].

Together with the risk of false negative and false positive results, RDTs could miss double infections and are not able to quantify the parasitemia and distinguish among the parasitic stages [31].

However, based on their results and the scientific literature in the topic, Grobush and colleagues [31] conclude that the combination of HRP2 for *P. falciparum* detection and pLDH antigens for *P. vivax* detection might be the best way to realize a reliable RDT for malaria diagnosis. In this light, the sensitivity in detecting species other than *P. falciparum* and *P. vivax* is very low [25,33].

The observations reported by these authors meet with the Guideline for the laboratory diagnosis of malaria by Bailey et al. in 2013 [34], intended for UK and applicable to other non-endemic areas, suggesting the use of RDTs to confirm the presence or absence of *P. falciparum* assessed by microscopy, particularly when an inexperienced observer is involved in the diagnosis. However, for the reasons already exposed, they cannot substitute microscopy and their use is not recommended for following the response to antimalarial treatment. Furthermore, the currently available RDTs are not able to detect *P. knowlesi* [34,35].

RDTs have been proposed to be used as self-diagnosis technique for high-risk groups, such as travellers in endemic areas after appropriate instructions and training to allow prompt treatment and avoid over-diagnosis of malaria on-site; although some recent results are encouraging, this application is still controversial [28,36].

## 4. Molecular Assays

Although microscopy is still the reference method because of the reasons described above, and RDTs provide valid support for diagnosing malaria, molecular assays have been proposed as a confirmatory method. In particular, they are crucial in cases of sub-microscopic parasitemia and when morphologic characteristics overlap, and/or when parasite morphology has been altered by drug treatment or improper storage of the sample [17]. CDC suggests the use of the real-time PCR assay developed by Rougemont et al., 2004, and when a mixed infection is suspected, a nested-PCR assay by Snounou et al., 1993, which could improve the resolution [17].

In Europe, molecular methods have been largely evaluated [37,38,39,40,41,42,43,44,45,46,47,48,49,50,51,52,53,54,55,56].

Overall, nucleic acid amplification tests (NAATs) are at least 10-fold more sensitive than microscopy [34]. The limit of detection for NAATs is approximately 0.2–6 parasites per μL of blood, depending on the assay and the species of *Plasmodia* involved [57]. 

The first target considered—and it is still used as a reference target—is the 18S-rRNA gene, present in 5–8 copies per *Plasmodium* genome. In particular, this reference target includes a genus-specific sequence of approximately 1.2 kb containing all the *Plasmodium* human-infecting species-specific sequences, which have been characterized and sequenced [42,53,58,59].

Newly developed NAATs include additional target genes, such as mitochondrial DNA (mtDNA), which allows the detection of all human malaria species together with 18S-rRNA, and other targets focusing on single species detection, such as *P. falciparum* stevor multigene family, telomere-associated repetitive element, and *P. vivax* Pvr64 sequence [60]. The 18S-rRNA gene exists in the chromosomal genome 5–8 copies depending on the strain; mitochondrial DNA exists in about 20 copies in the mitochondrial organelle. In the early ring stage, *P. falciparum* parasite has one mitochondrion, whereas mature gametocytes have 4–8 mitochondrial organelles [42]. In a study performed in 2013, mitochondrial PCR demonstrated to have sensitivity non-inferior to that of 18S-PCR, and interestingly, the short product size allows easy full-length sequencing [42]. The different features of these different targets were used to observe the presence of plasmodial DNA in follow-up samples post-treatment, and to determine the proportion of positive PCRs due to gametocytes in an observational study of the same research group using PCR assays targeting the var acidic terminal sequence (*varATS*) gene, located on the chromosomal genome, and *cytochrome b* (*cytb*) on the mitochondrial genome. The authors assumed that, as previously demonstrated, most individuals with asexual parasites also have sub-microscopic gametocyte carriage. Interestingly, *cytb* PCR detection in follow-up samples later than *varATS* PCR may be due to the detection of gametocytes, as hypothesized by the authors. However, based on their observations, the authors concluded that it is unclear whether the DNA detected after treatment originated from residuals of destroyed parasites or live gametocytes [61].

In all the studies cited in Table 2, the evaluated molecular assays, as expected, demonstrated better performance than conventional methods. Besides higher sensitivity, specificity, and accuracy, they allow to detect *Plasmodia* not only at the genus level, but at the species level too [37,38,39,40,41,42,43,44,45,46,47,48,49,50,52,53,54,55,56] and they allow species identification in cases of mixed infections [37,56]. This is particularly evident in non-*P. falciparum* infections with low parasite density and it is important to *P. malariae* and *P. ovale* malaria because the sensitivity of RDTs can be very low [44].

**Figure 4:**
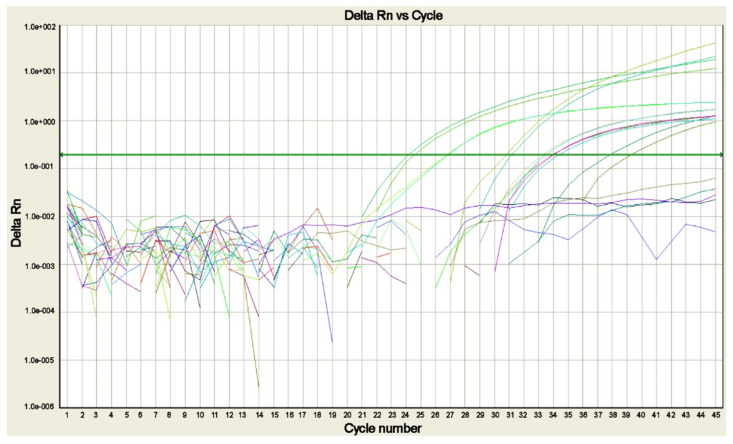
Real-time PCR amplification plot for the search of *Plasmodia* DNA in blood samples of cases of suspected malaria. The plot shows the amplification of *P. falciparum*, *P. malariae*, *P. ovale curtisi*, *P. ovale wallikeri*, and *P. vivax* positive controls and of the sample positive for *P. falciparum*, each tested in duplicate. The green line corresponds to the threshold (picture by A. Calderaro, Department of Medicine and Surgery, University of Parma, Parma, Italy).

Microscopy is the gold-standard method, and that cannot be avoided; however, different laboratories include in their workflows the molecular assays in ways that best suit their needs. In some laboratories, for example, the molecular assay is performed when species identification is problematic or in cases of strong suspicion of malaria with negative results by conventional methods [52,55]. Rougemont and colleagues [52] affirm that the development of automated PCR platforms and the unavailability of skilled microscopists will make molecular diagnosis more appealing at a reasonable cost, even or especially during nights and weekends. Conventional PCR has been the starting point for more sensitive, specific, and complex assays, such as nested-PCR and the application of Southern blot for the identification of *Plasmodia* species [37,40,49,50]. New PCR protocols evolving from conventional PCR are always in development to simplify the analysis and to reduce the possibility of contamination. As a matter of fact, conventional PCR (including the nested-PCR) is labor-intensive, time consuming, susceptible to cross-contamination by PCR products, and vulnerable to false-positive results [40,48]. This problem could be tackled by adopting several precautions [40] or developing more “safe” techniques, such as real-time PCR (Figure 4).

Real-time PCR assays are highly sensitive and specific, and far less labor-intensive. They are performed in a closed system where post-PCR handling is not required and limit the possibility of contamination together with a good rapidity, although they cannot be strictly considered a rapid technique for the initial diagnosis of malaria requiring more than 1 h [52,53]. Furthermore, as they allow DNA quantification too, their use was proposed to potentially determine the reduction of the parasite load to monitor the therapeutic efficacy [52]. In a recent study, besides the successful evaluation of two commercial kits for *Plasmodia* detection, the correlation between real-time PCR’s cycle threshold and parasitemia was also assessed, as previously performed [62]. Unsatisfactory and weaker results were obtained, maybe because of different storage and carriage conditions [63].

Among the different available molecular techniques, a faster and simpler method than real-time PCR for the diagnosis of malaria is a real-time quantitative nucleic acid sequence-based amplification (QT-NASBA) assay evaluated in Amsterdam [51,54] that proved to be a sensitive and specific technique useful for both the detection and the quantification of *Plasmodia* 18S-rRNA for diagnostic purposes and epidemiological and drug studies [51,54].

One of the most recent evolutions of DNA amplification for malaria diagnosis is the development of commercial assays based on the DNA loop-mediated isothermal amplification (LAMP) that reduce the analysis time within the 2-h delay recommended for the diagnosis and ensure a simple technical process and a high sensitivity [43,44,47]. An interesting result was obtained in a 2017 study evaluating a commercial LAMP assay (Pan and Pf LoopAMP^®^-Eiken Chemical Co., Tokyo, Japan) for the detection of *P. ovale* malaria. The LAMP results were discordant in 2.6% of samples as compared to the nested-PCR used as a reference method: it remains to be determined whether there were false positives by LAMP, or false negatives with very low parasitemia by nested-PCR, as already reported [64]. The authors were satisfied by the assay’s performance. They judged it as a useful tool for malaria control and elimination programs and in targeting returning travellers from *P. ovale* endemic areas [44]. In the same study, an evaluation of the LAMP results by the naked eye in comparison with the use of turbidimeter was performed; there was good correspondence, as deemed by the authors [44]. However, for such an assay [47], the target sequence is not declared, and this remains a bias for its use in the practice and its comparison with other assays.

Dakić and colleagues [38] encourage the use of molecular assays, especially in non-endemic settings, as a complementary method to microscopy, particularly in cases of low parasitemia and for species determination, taking into account that most instances of misdiagnosis occur in cases of malaria by *Plasmodia* other than *P. falciparum*. Although the improved sensitivity is evident, their adoption and inclusion in the workflow should be deeply evaluated.

It cannot be ignored that they detected the parasitic DNA while not distinguishing among DNA belonging to live parasites, residual DNA of destroyed asexual blood stage parasites, and circulating gametocytes which can remain in sub-microscopic quantities after successful therapy, thereby risking false positives due to the persistence of DNA after a malaria episode’s resolution, and as a consequence, unnecessary malaria treatment interventions [38,45,46]. However, a control experiment performed in an animal model [65] demonstrated the clearance of parasite DNA from blood within 48 h after malaricides treatment; thus, it can be inferred that *Plasmodium* DNA detected in blood is probably a sign of active infection, even if no parasites are detected by microscopy [50]. Further disadvantages are the requirements for a sophisticated laboratory setting and trained operators, and the higher costs [45]. The wide spread of different molecular assays for the diagnosis of malaria, often developed in-house, laid the foundations in 2008 for the establishment by WHO of an International Standard for *Plasmodium falciparum* DNA for (NAT)-based assays that can be used for quality control and in the determination of the analytical sensitivity of different assays [66].

These considerations strengthen the need to carefully apply molecular techniques to the diagnosis of malaria.

One of the main current challenges is the detection of *P. knowlesi* in travellers with suspected malaria returning from Southeast Asia. The detection of *P. knowlesi* is mandatory, since the infection can be fatal if not treated promptly; however, its identification by microscopy is particularly difficult because of the morphological resemblance of early trophozoites to *P. falciparum* and later erythrocytic stages to *P. malariae* [41]. In this light, the inclusion of molecular assays in the malaria diagnostic workflow in Europe became essential, and as reported in Table 2, it was applied successfully by different authors [41,63].

**Table 2:** Molecular assays field evaluated by European diagnostic laboratories.

Evaluated Assay	Type of Amplification	Target	Country	Samples/ Patients Tested	Period	Reference Test	Performance/ Agreement with Reference Test	Reference
In-house species-specific PCR	Nested PCR	18S rRNA	Spain	192 samples/patients with suspected malaria	1997–1998	Microscopy	12.4% more malaria cases detected by PCR	[37]
In-house genus-specific PCR	Conventional genus-specific PCR	18S rRNA	Italy	101 samples/ patients with suspected malaria	1994–1999	Microscopy	Sensitivity 100% Specificity 100%	[49]
species-specific PCR by [67] and by [68]	species-specific Southern blot						Agreement 94%	
In-house species-specific PCR by [58]	Nested PCR	18S rRNA	Poland	216 patients with suspected malaria	/	Microscopy	Agreement 83.8%	[50]
In-house genus-specific PCR	QT-NASBA	18S rRNA	The Netherlands	113 patients with suspected malaria	4 months	Microscopy	Sensitivity 100% Specificity 94% Agreement 94.7%	[51]
In-house genus-specific qPCR	TaqMan	18S rRNA	Switzerland	97 samples/ 66 from patients with suspected malaria + 31 from patients with known *Pf* malaria	2002–2003	Microscopy	86% agreement	[52]
In-house species-specific qPCR							71% agreement	
In-house genus-specific PCR+ species-specific PCR (*Pf*, *Pv*, *Po*)	TaqMan	18S rRNA	Italy	122 samples/patients with suspected malaria	/	18S rRNA nested PCR	Sensitivity 100% Specificity 100%	[53]
In-house species-specific PCR	QT-NASBA	18S rRNA	The Netherlands	79 samples of patients with malaria	/	Microscopy	Perfect agreement (evaluated by Cohen’s Kappa coefficient)	[54]
*Pf* qReal Time-PCR by [69]	Sybr Green	*Pf* CoxI gene	France	192 patients with suspected malaria	2005–2007	Microscopy +RDT	93.3% agreement	[55]
In-house genus-specific qPCR		*Plasmodium* mitochondrial sequence					99% agreement	
In-house species-specific qPCR		18S rRNA					98% agreement	
Genus-specific qPCR by [52] with modifications	TaqMan	18S rRNA *Plasmodium* gene	Belgium	351 samples	1995–2009	Microscopy	8.3% cases detected only by PCR	[56]
In-house species-specific PCR		18S rRNA specific genes					1.3% species identification only by PCR	
In-house species-specific PCR by [70]	Seminested PCR	18S rRNA	Italy	1226 patients with suspected malaria	1998–2003	Microscopy	Sensitivity 100% Specificity 100% PPV 100% NPV 100%	[40]
In-house species-specific PCR for *Poc* and *Pow*	TaqMan	18S rRNA	Italy	31 samples from patients with *P.ovale* malaria	/	18S rRNA nested PCR	100% agreement	[39]
Genus-specific PCR by [58]	TaqMan	18S rRNA	Norway	135 samples/patients with suspected malaria	2006–2011	Nested SSU rRNA PCR	93% agreement	[42]
Genus-specific PCR by [71]		Mitochondrial DNA sequence					97% agreement	
Species-specific PCR [72]		18S rRNA					87% agreement	
In-house genus-specific PCR + species -specific PCR	TaqMan	18S rRNA (including *Pk*, *Poc* and *Pow*)	Italy	398 samples/ patients with suspected malaria	2000–2012	Microscopy	6.3% species identification only by PCR:1.5% samples disagreement with microscopy	[41]
Genus-specific qPCR [52]	TaqMan	18S rRNA *Plasmodium* gene	Serbia	109 samples/patients with suspected malaria	2010–2013	Microscopy	95.5% agreement	[38]
Species-specific qPCR by [53] (for *Pf*, *Pv*, *Po*) and by [52] (for *Pm*)		18S rRNA specific genes					73.3% agreement	
Pan and *Pf* LoopAMP^®^ (Eiken Chemical Co.)	LAMP	Mitochondrial DNA sequence	Switzerland	210 samples/patients with suspected malaria	March–October 2012	Microscopy	Sensitivity 100% Specificity 97.5% PPV 91.5% NPV 100%	[43]
						18S rRNA qPCR	Sensitivity 100% Specificity 100% PPV 100% NPV 100%	
Pan and *Pf* LoopAMP^®^ (Eiken Chemical Co.)	LAMP	Mitochondrial DNA sequence	Spain	427 samples: 29 *Po* positive samples+ 398 negative samples stored	2014–2016	Nested SSU rRNA PCR	Sensitivity 100% Specificity 97.2% PPV 72.5% NPV 100%	[44]
Genus-specific PCR and Species-specific PCR (FTD Malaria, Fast-Track Diagnostics^®^)	TaqMan	/	Spain	250 patients: 86 with suspected malaria+ 164 asymptomatic immigrants from endemic areas	2015–2017	In-house genus-and species-specific PCR	Sensitivity 96% Specificity 97.4% PPV 93.6% NPV 98%	[45]
Species-specific PCR by RealStar Malaria S&T PCR Kit 1.0	TaqMan	*Plasmodium* spp. DNA (including *Pk*)	Germany	179 samples positive by microscopy and genus-specific PCR	April–December 2017	Microscopy+ genus-specific PCR	Sensitivity 95.1%	[46]
Genus-specific PCR and Species-specific PCR (FTD Malaria, Fast-Track Diagnostics^®^)							Sensitivity 96.8%	
In-house species-specific duplex PCR for *Poc* and *Pow* by [73]	TaqMan	18S rRNA	Germany	77 samples/patients with *P.ovale* malaria	2010–2019	/	100% agreement among the 2 evaluated assays	[63]
In-house species-specific singleplex PCR for *Poc* and *Pow* described by [74] and [39]								
Alethia assay (Meridian Bioscience)	LAMP	Undeclared target: segments of the *Plasmodium* genome	France	331 samples/patients with suspected malaria	2017–2018	Real-time PCR	Sensitivity 97.3% Specificity 99.6% PPV 94.8% NPV 99.8%	[47]
In-house *Pfhrp2* PCR	TaqMan	*Pfhrp2* gene	United Kingdom/ Switzerland/ Portugal	50 DNA samples from suspected *Pf* patients from Eritrea	/	Conventional qPCR	Sensitivity 100%	[48]
In-house *Pfhrp3* PCR		*Pfhrp3* gene					Specificity 100%	

Performance: calculation of diagnostic sensitivity, specificity, positive predictive value, and/or negative predictive value. *CoxI:* mitochondrial cytochrome C oxidase. *Pf: Plasmodium falciparum, Po: P. ovale, Pv: P. vivax, Pm: P.malariae, Poc: P. ovale curtisi, Pow: P. ovale wallikeri, Pk: P. knowlesi. PCR:* Polymerase Chain Reaction. PPV: Positive Predictive Value. NPV: Negative Predictive Value. LAMP: loop-mediated isothermal amplification. SSU: small subunit. QT-NASBA: Quantitative Nucleic Acid Sequence-Based Amplification. /: Not reported.

**Figure 5:**
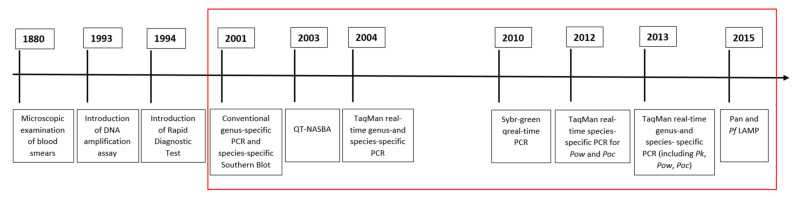
Milestones of the introduction of diagnostic assays for malaria (the red rectangle shows the milestones included in this review) [21,39,41,43,49,51,52,53,55,58,67].

A summary of the key features of, and the desired improvements for, microscopic examination, RDTs, and NAATs for the diagnosis of malaria, are reported in Table 3. Moreover, the milestones in the introduction of the methods currently used, since the discovery of malaria parasites in 1880 by microscopy [75], and herein described, are shown in Figure 5, highlighting that the novelties proposed in the last 22 years are improvements and evolutions of previously developed assays.

## 5. Other Diagnostic Methods

Together with molecular assays, other novel techniques have been developed for the diagnosis of malaria, particularly those detecting hemozoin [76,77,78,79,80,81] in both endemic and non-endemic areas, which can be mutually exported. The starting point is the assumption that the detection in a patient’s leukocytes of hemozoin, generated through the digestion of the globin part of hemoglobin by Plasmodia, is indicative of malaria infection [76]. Hänscheid and colleagues [77,82] have developed a flow-cytometry assay by using an automated full blood counts (FBC) instrument that, taking advantages from the anisotropic properties of hemozoin, allows to detect the Plasmodium sp. pigment in those laboratories where FBC is routinely performed. Although promising, if applied in addition to conventional methods, this approach still requires extensive field evaluation [82]. 

In 2010, Mens et al. [76] evaluated the magneto-optical technology (MOT) exploiting the paramagnetic features of hemozoin. When the samples are submitted to a magnetic field, the hemozoin crystals, if present, align with the magnetic field. A laser-based instrument able to quantify this phenomenon allows to understand whether hemozoin is present or not in a sample. The results obtained demonstrated a performance not yet at a competitive level compared to other diagnostic tests [76]. A technical improvement in the magneto-optical detection of hemozoin crystals has been recently proposed by Arndt et al. [78] in Papua New Guinea. The authors hope it will be used in other settings too. The novel diagnostic technique named rotating-crystal magneto-optical detection (RMOD) maximizes the MO signal, rapidly providing a measurement of the magnetically induced linear dichroism of hemozoin. Furthermore, RMOD demonstrated to be able to quantify the amount of the pigment in a sample. However, RMOD, by revealing the presence of residual hemozoin, is not able to discriminate between current and previous infections. The authors affirmed that this limitation is expected to be reduced in low-transmission settings. Moreover, in the current state of development, RMOD cannot distinguish between parasite species in P. falciparum and P.vivax co-endemic settings. Thus, according to the authors’ conclusions, this technique requires further evaluation and potential further improvements for both endemic and non-endemic settings [78].

The magnetic susceptibility of hemozoin has led to the development of innovative detection methods based on nuclear magnetic resonance (NMR) and on magnetic resonance relaxometry (MRR) ([79,80], respectively). Gupta et al., in 2020 [80], proposed a portable banchtop assay based on NMR that turned out to be sensitive, easy to handle, cost-effective, and able to work with only a small sample volume. In the same year, Di Gregorio et al. [79] developed an MRR assay that appears to be an efficient tool for the detection of P. falciparum-parasitized RBC and that could be useful to assess the effects of dihydroartemisinin and chloroquine.

The detection of hemozoin in RBC parasitized by P. falciparum has been investigated also by using a novel photoacustics (PA) excited surface acoustic wave (SAW) sensor [81]. The authors demonstrated the good potential of a PA-SAW sensor in the diagnosis of malaria at early stages and at a concentration of 1%. They aimed to improve the performance of the developed technique and to extend its use to other parasite species. 

In conclusion, therefore, the described novel techniques that search for hemozoin are not yet tools applicable to the diagnosis of malaria, but they could be promising solutions, after improvements, for future diagnostic systems.

**Figure 6:**
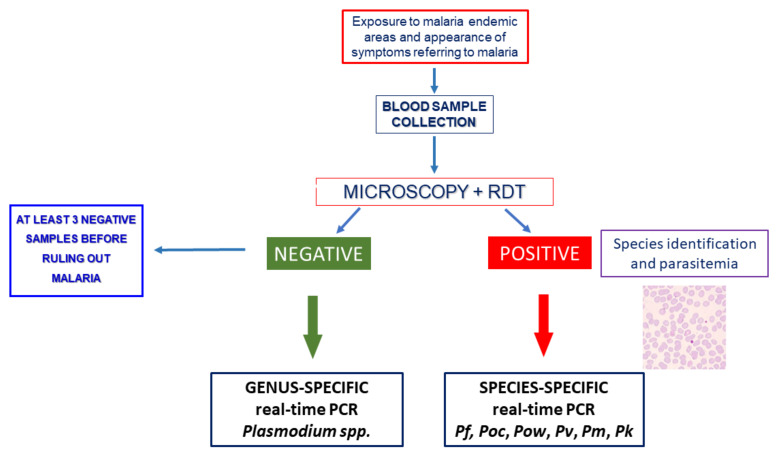
A diagnostic algorithm for malaria for non-endemic areas. *Pf: Plasmodium falciparum, Pv: P. vivax, Pm: P. malariae, Poc: P. ovale curtisi, Pow: P. ovale wallikeri, Pk: P. knowlesi.* PCR: polymerase chain reaction. RDT: rapid diagnostic tests.

In Figure 6, an algorithm for the laboratory diagnosis of malaria is proposed for both endemic and non-endemic areas, on the basis of that reported by WHO, based on microscopic examination and rapid diagnostic tests [83].

## 6. Conclusions

Malaria is a rare diagnosis in Europe, but it is a medical emergency. A travel history is the key when malaria is suspected, and it is mandatory in patients with fever. There are no specific clinical signs or symptoms of malaria, although fever is seen in almost all non-immune patients. Migrants from malaria-endemic areas may have few symptoms.

Malaria diagnostics should be performed immediately on suspicion of malaria, and the gold-standard is microscopy of Giemsa-stained thick and thin blood films. The quantification of malaria parasites can be used to make clinical management decisions and to monitor responses to treatment. Microscopy diagnosis is prone to human error, owing to its subjective nature. An inherent weakness of microscopy is the dependence on morphological features when *Plasmodium* species are being distinguished. Even under ideal conditions, reliable distinction of the infecting *Plasmodium* species can be very difficult, if not impossible. Particularly, *P. vivax* and *P. ovale* cannot always be easily differentiated based on morphology; distinguishing *P. knowlesi* from *P. malariae* can be very challenging; *P. ovale wallikeri* and *P. ovale curtisi* are morphologically identical; *P. cynomolgi* is morphologically indistinguishable from *P. vivax*; and *P. simium* and *P. brasilianum* cannot be distinguished by microscopy from *P. vivax* and *P. malariae*, respectively. The limit of detection is also not ideal, because sub-microscopic asymptomatic individuals with low parasitemia remain undiagnosed and untreated, and also enable the transmission cycle to continue in the community. 

A RDT may be used in parallel, but should not replace microscopy [20]. It is a fast and affordable method for malaria diagnosis; the personnel training required is much less intensive as compared to microscopy and PCR. However, it does not allow for the quantification of parasitemia, and consequently, monitoring therapeutic effectiveness is difficult [20,84]. Microscopy remains the gold-standard technique for diagnosis but RDTs, originally limited to endemic areas and returning travellers from endemic areas, are now more widely used as a complement to microscopy [85].

Molecular methods have demonstrated to be more sensitive and specific than microscopy, allowing the detection of missed cases and correctly identifying the species of Plasmodia of medical interest, with the final result of improving the early diagnosis of all cases of imported malaria [14,20]. However, their application should be deeply evaluated because of the risk of false positives due to the persistence of DNA after malaria episodes resolve [38,45,63].

The proposed algorithm takes into account these observations and the essential contribution of the genus- and species-specific DNA amplification assays for accurate diagnosis of malaria.

According to WHO Global Technical Strategy for Malaria 2016–2030 [8], the future direction for the diagnosis of infectious diseases, including malaria, in both endemic and non-endemic settings, is the development of point-of-care testing (POCT) in response to the request for rapid diagnosis, together with “on-site” results, which would be helpful for prompt and accurate treatment and for preventing the transmission of infectious diseases [86]. Several research groups developed new generation assays, or adapted pre-existing assays to smart devices. Furthermore, at present, efforts are being made to support POCT by using devices derived from innovations in the field of Internet of Medical Things (IoMT), offering wireless-based operations and connectivity of such devices with medical centers [86]. 

This review showed that diagnostic laboratories in malaria non-endemic settings provide excellent diagnosis of malaria, especially regarding the detection of P. falciparum. 

Despite the limitations of current diagnostic methods, they continue to play important roles in dealing with the current global malaria situation, including decreasing its incidence. 

Diagnostic tools are critical for ensuring the appropriate care for each patient, and in this light, the development of numerous innovations continues. 

## Figures and Tables

**Table 1:** Rapid diagnostic tests field evaluated by European diagnostic laboratories.

Evaluated Assay	Antigen Detected	Country	Samples/Patients Tested	Period	Reference Test	Performance/ Agreement with Reference Test	Reference
ParaSight-F (Becton Dickinson)	*Pf*HRP2	Spain	206 samples/ 169 patients with suspected imported malaria	1998–1999	Microscopy PCR	Sensitivity 76.8% Specificity 76.7% PPV 65.4% NPV 85.2% Sensitivity 67.8% Specificity 77.1% PPV 70.4% NPV 75%	[30]
ICT Malaria *Pf*/*Pv* (Amrad)	*Pf*HRP2/ pan-aldolase				Microscopy PCR	Sensitivity 81.2% Specificity 97.4% PPV 92.8% NPV 92.5% Sensitivity 68.4% Specificity 97.1% PPV 92.8% NPV 85%	
OPTIMAL^®^ (Flow Incorporated)	*Pf*LDH/pLDH				Microscopy PCR	Sensitivity 66.1% Specificity 81.5% PPV 62.1% NPV 84% Sensitivity 62.7% Specificity 84.4% PPV 71.2% NPV 78.6%	
*Para*Sight F (Becton Dickinson)	*Pf*HRP2	United Kingdom	160 patients with suspected malaria	1994–1996	Microscopy	Sensitivity 93.3% Specificity 98.3% PPV 95.6% NPV 97.4%	[27]
ParaSight-F (Becton Dickinson)	*Pf*HRP2	Italy	139 samples/patients with suspected imported malaria	1994–1999	Microscopy	Sensitivity 50–100%	[29]
OPTIMAL^®^ (Flow Incorporated)	*Pf*LDH/pLDH					Sensitivity 0–100%	
ICT Malaria *Pf*^®^ (ICT Diagnostics)	*Pf*HRP2/ pan-aldolase	Germany	231 patients with suspected imported malaria	10 years	Microscopy	Sensitivity 92.5% Specificity 98.3% PPV 94.2% NPV 97.8%	[28]
OPTIMAL^®^ (Flow Inc.)	*Pf*LDH/pLDH					Sensitivity 88.7% Specificity 99.4% PPV 97.9% NPV 96.7%	
ParaSight-F (Becton Dickinson)	*Pf*HRP2	Germany	1073 samples/ 850 patients with suspected imported malaria	1998–2001	Microscopy	Sensitivity 95.1% Specificity 97.1%	[31]
ICT Malaria *Pf* (ICT Diagnostics)	*Pf*HRP2					Sensitivity 90.6% Specificity 99.4%	
ICT Malaria *Pf*/*Pv* (ICT Diagnostics)	*Pf*HRP2/pan-aldolase					Sensitivity 97.7% Specificity 98.8%	
OPTIMAL^®^ (Flow Inc.)	*Pf*LDH/pLDH					Sensitivity 76.2% Specificity 99.7%	
Now Malaria dipstick (Binax)	*Pf*HRP2/ pan-aldolase	France	413 patients with suspected imported malaria	2002–2004	Microscopy/ QBC	*Pf* Sensitivity 96.4% Specificity 97% PPV 84% NPV 99.4%	[32]
						Non-*Pf* Sensitivity 66.7% Specificity 100% PPV 100% NPV 98.2%	
Palutop+4 (All.Diag)	*Pf*HRP2/ *Pv*pLDH/pLDH	Belgium	613 samples selected from international travellers	1995–2008	Microscopy corrected by PCR	*Pf* Sensitivity 85.1% Specificity 96.9% *Pv* Sensitivity 66% Specificity 100% *Po* Sensitivity 5.5% *Pm* Sensitivity 32% *Po/Pm* Specificity 100%	[33]
ICT Malaria *Pf*/*Pv* (Amrad)	*Pf*HRP2/ pan-aldolase	Switzerland	2139 adult patientswith malaria	1999–2007	Microscopy	Full agreement: 94%	[15]
OPTIMAL^®^ (Diamed)	*Pf*LDH and pLDH						
Now ICT Malaria (Binax)	*Pf*HRP2/ pan-aldolase	France	1311 patients with suspected malaria	2006–2008	Microscopy	Sensitivity 93% Specificity 97%	[25]
Core Malaria Pan/*Pv*/*Pf* (Ivagen)	*Pf*HRP2/pLDH/ *Pv*LDH					Sensitivity 94% Specificity 96%	
Palutop+4 (All Diag)	*Pf*HRP2/pLDH/ *Pv*LDH					Sensitivity 94% Specificity 97%	
Optimal-IT (Diamed)	*Pf*LDH/pLDH					Sensitivity 83% Specificity 99%	
VIKIA Malaria Ag *Pf*/Pan™	*Pf*HRP2/ pan-aldolase	France	155 patients with suspected malaria	2011	Microscopy corrected by PCR	*Pf* or mixed Sensitivity 98% Specificity 93.1% PPV 87.3% NPV 98.9%	[21]
						Non-*Pf* Sensitivity 60% Specificity 100% PPV 100% NPV 95.7%	
SD Bioline Malaria Ag *Pf*/Pan	*Pf*HRP2/pLDH	Greece	955 samples/patients with suspected malaria, residents around the case’s house and residents in regions where autochthonous cases occurred	2012	Microscopy	Sensitivity 97.3% Specificity 99.4% PPV 86.1% NPV 99.9%	[12]
					PCR	Sensitivity 95.6% Specificity 100% PPV 100% NPV 99.8%	
ICT Malaria Combo Cassette (ICT Diagnostics)+ SD Bioline Malaria Ag *Pf*/Pan malaria (Standard Diagnostics)	*Pf*HRP2/ pan-aldolase *Pf*HRP2/ pLDH	France	446 samples/ patients with imported malaria	2006–2018	Microscopy (from 2017 a LAMP assay-Alethia Malaria kit, Meridian^®^-was associated)	Full agreement: 99.3%	[26]

Legend: Performance: calculation of diagnostic sensitivity, specificity, positive predictive value and/or negative predictive value. *Pf: Plasmodium falciparum. Po: P. ovale.* *Pv: P. vivax. Pm: P. malariae.* QBC: quantitative buffy coat. PCR: Polymerase Chain Reaction. PPV: Positive Predictive Value. NPV: Negative Predictive Value.

**Table 3:** Key features and future desired improvements of the methods for the diagnosis of malaria.

Key Features	Microscopic Examination	Rapid Diagnostic Tests (RDT)	Nucleic Acid Amplification Tests (NAATs)	Future Desired Improvements
Sensitivity	50–200 parasites/µL blood	200–2000 parasites/µL blood	0.2–6 parasites/µL blood	RDT sensitivity increasing
Specificity	98%	68.4–100% (depending on the assay and the species)	94–100%	RDT discrimination capability for *Plasmodia* species other than *Pf*
Identification of parasites at species level	Yes (Not distinguish between *Poc* and *Pow*)	Not distinguish among *Pv/Poc/Pow/Pm.* Not able to detect *Pk*	Yes (distinguish also between *Poc* and *Pow*)	RDT discrimination capability increasing and introduction of *Pk* identification
Detection of mixed infections	Low capability	Low capability	High capability	RDT discrimination capability increasing
Identification of parasites stages	Yes	No	No	Improvement of the skills of the microscopists in non-endemic settings
Turn-around-time	Rapid (suitable to be performed in emergency)	Rapid (suitable to be performed in emergency)	Rapid	NAAT suitable to be performed in emergency
Determination of parasitemia	Yes	No	No	NA
Monitoring of patients with malaria including follow-up during specific therapy	Yes	No	No	NA
Automatization	No	No	Yes	Extension to a wide number of laboratories of automatized tools
Instrument required	Yes	No	Yes	NA
Trained personnel	Yes	No	No	Improvement of the skills of the microscopists in non-endemic setting
Costs	Low	Moderate	High	Reduction of the costs particularly for endemic settings

*Pf: Plasmodium falciparum. Pv: P. vivax. Pm: P. malariae. Poc: P. ovale curtisi. Pow: P. ovale wallikeri. Pk: P. knowlesi*. NA: not applicable.

## Data Availability

Not applicable.
